# Operational Modes Detection in Industrial Gas Turbines Using an Ensemble of Clustering Methods

**DOI:** 10.3390/s21238047

**Published:** 2021-12-01

**Authors:** Mina Bagherzade Ghazvini, Miquel Sànchez-Marrè, Edgar Bahilo, Cecilio Angulo

**Affiliations:** 1 Computer Science Department, Intelligent Data Science and Artificial Intelligence Research Centre (IDEAI), Universitat Politècnica de Catalunya, 08034 Barcelona, Spain; mina.bagherzade.ghazvini@upc.edu; 2 Siemens Energy S.L., Slottsvägen 2-6, 612 31 Finspang, Sweden; edgar.bahilo_rodriguez@siemens.com; 3 Automatic Control Department, Intelligent Data Science and Artificial Intelligence Research Centre (IDEAI), Universitat Politècnica de Catalunya, 08028 Barcelona, Spain; cecilio.angulo@upc.edu

**Keywords:** artificial intelligence, ensemble of clusters, clustering, operational modes, gas turbine

## Abstract

Operational modes of a process are described by a number of relevant features that are indicative of the state of the process. Hundreds of sensors continuously collect data in industrial systems, which shows how the relationship between different variables changes over time and identifies different modes of operation. Gas turbines’ operational modes are usually defined regarding their expected energy production, and most research works either are focused a priori on obtaining these modes solely based on one variable, the active load, or assume a fixed number of states and build up predictive models to classify new situations as belonging to the predefined operational modes. However, in this work, we take into account all available parameters based on sensors’ data because other factors can influence the system status, leading to the identification of a priori unknown operational modes. Furthermore, for gas turbine management, a key issue is to detect these modes using a real-time monitoring system. Our approach is based on using unsupervised machine learning techniques, specifically an ensemble of clusters to discover consistent clusters, which group data into similar groups, and to generate in an automatic way their description. This description, upon interpretation by experts, becomes identified and characterized as operational modes of an industrial process without any kind of a priori bias of what should be the operational modes obtained. Our proposed methodology can discover and identify unknown operational modes through data-driven models. The methodology was tested in our case study with Siemens gas turbine data. From available sensors’ data, clusters descriptions were obtained in an automatic way from aggregated clusters. They improved the quality of partitions tuning one consistency parameter and excluding outlier clusters by defining filtering thresholds. Finally, operational modes and/or sub-operational modes were identified with the interpretation of the clusters description by process experts, who evaluated the results very positively.

## 1. Introduction

Industrial gas turbines are energy systems widely used in industries such as power plants, transportation, aerospace, and off-shore platforms [1,2,3,4]. As an important component in industry, associated key issues are maintenance and performance enhancement [5,6,7,8,9,10,11]. To reduce maintenance costs as well as to improve the performance of gas turbines, condition monitoring solutions are deployed. Condition monitoring is a maintenance approach that predicts machine health and safety through the combination of machine sensor data and machine monitoring software. Hence, by extracting sensors information of gas turbine historical data, software-based diagnostic and prognostic analysis can be implemented [12].

On one hand, prediction of performance deterioration is an important and complex task, as the operational condition of gas turbines is not static and due to the uncertainty of ambient conditions. *Prognostic techniques* for performance deterioration can be categorized in two types: *model-based prognostics* and *data-driven methods* [13,14,15,16,17]. On the other hand, *diagnostic analysis* for anomaly detection and fault conditions classification has attracted significant attention. Gas turbine dynamics complexity and non-linear behavior could lead to unexpected shutdowns, over-heating, over-speed during operations and system disturbances due to fault or load fluctuations [18]. Different diagnostic techniques have been developed, which can be summarized into three types, *model-based diagnostics*, *data-driven approach* and *hybrid methods*, which are a combination of the first two approaches [19,20,21,22].

In particular, our interest is focused on *data-driven approaches*, which rely on the accuracy and completeness of the gas turbine’s historical data. By extracting behavior patterns and experience from signals, system conditions should be distinguished. Due to fast development of computational technologies and sensors, most of the prognostic and diagnostic systems in industries tend to use signal processing techniques. Existing data-driven methods or signal processing-based techniques include statistical methods [23], artificial neural networks (ANNs) [24] and other soft computing techniques [25].

A few works exist on trying to discover the operational states of a process, such as the work in [26], where a Gaussian kernel fuzzy C-means clustering algorithm was used to detect eight fault types, but the number of different states is a priori known. Other works in the literature obtained predictive models to classify new data into well-defined operational states using several techniques, such as Gaussian mixture models [27] or learning vector quantization (LVQ) and support vector machines (SVM) [28] or those based on the deep belief network optimization algorithm [29]. There are some other studies on determining operational modes that focused on detecting one mode of operation, for example, steady state or partial load. Simon and Litt [30] developed a system that automatically extracts steady-state engine operating points from engine flight data. In [31], Davison described a technique that measures how close an engine is to steady state while operating. Ceils et al. [32] developed a signal analysis module that can determine the operating regimes of industrial gas turbines. Its use, however, is intended for monitoring and diagnostics of steady state operating conditions. Moreover, Reference [33] only analyzed the partial load of gas turbine. The paper [34] described a method to only detect the number of operational modes in baseline multivariate statistical process control data with a scale-based approach. All these studies on similar problems are compiled and summarized in Table 1, where the main characteristics of these research works are detailed, and their limitations are outlined to clearly describe the contextual scenario. From Table 1, it can be stated that most of similar works cannot solve the problem of identifying *unknown operational states/modes* of an industrial process from its sensor data. This is the main limitation or gap among existing approaches that is the issue that our proposed approach wants to solve.

Our work developed a methodology for identifying the operational modes of gas turbines, without any a priori bias in the type or number of operational modes being searched for. In general, it is assumed that operational modes include startup, shutdown, steady state, partial loads and full load states. Our proposed methodology is not only able to detect all these general behaviors, but it can also extract some hidden knowledge, such as sub-operating modes, or other non-energy related modes. By using an ensemble of clustering techniques, aggregated clusters can be more consistent than single clustering approaches by defining a consistency rate. This consistency rate can tune the number of consistent clusters obtained, and hence, once described and interpreted, the operational modes can be more precise. By choosing a low value, the method can detect the general operational modes and when increasing it, some sub-modes can emerge. Additionally, the outlier and unnecessary clusters can be automatically discarded along with the filtering method defined in our research. Furthermore, clusters’ description is generated automatically through the analysis of the conditional distribution of relevant variables regarding the different clusters. This description can be easily interpreted by the experts to identify the operational modes discovered.

The main contributions of this study can be summarized as follows:

- A general data-driven methodology to detect and identify *unknown operational states or operational modes* from real-time sensor data of any industrial process is proposed.
- The proposal of using an ensemble of partitions, i.e., set of clusters, and a *filtering method* that, using a *filtering threshold*, enables the detection of main consistent clusters and the discarding of outlier clusters.
- The *filtering method* can tune the number of general operation modes and sub-modes of the industrial process obtained.
- The automatic generation of a description of the final aggregated clusters, which, after interpretation from the experts, becomes a set of identified operational modes.
- A practical case study in the gas turbine process illustrates the applicability of the approach, which leads to determine the gas turbine operational modes and operational sub-modes.

### 1.1. Industrial Gas Turbines

Industrial gas turbines (GT) are combustion engines for power generation used in several industrial sectors to provide mechanical power, including power plants and offshore platforms [1]. In terms of industries profitability, gas turbines play a dominant role in aircraft, transportation, power generation, and also oil and gas exploration and production [4]. GTs are popular because of their many attractive properties, including high power to weight ratios, ease of installation, faster start-up times, flexibility, and environmental friendliness in the form of low pollution products [2].

Gas turbines are classified based on their structure, application, and power generation, from micro-gas turbines with a power range of 20 KW to 350 KW, small gas turbines for simple cycle applications, with output power ranging between 0.5 MW and 2.5 MW and efficiency rates of 15% to 25% up to heavy-duty frame-type gas turbines with output power of 3–480 MW, and efficiency of 30–46%. During this study, medium-sized industrial gas turbines are considered, which are used in generating power in power plants.

**Figure 1:**
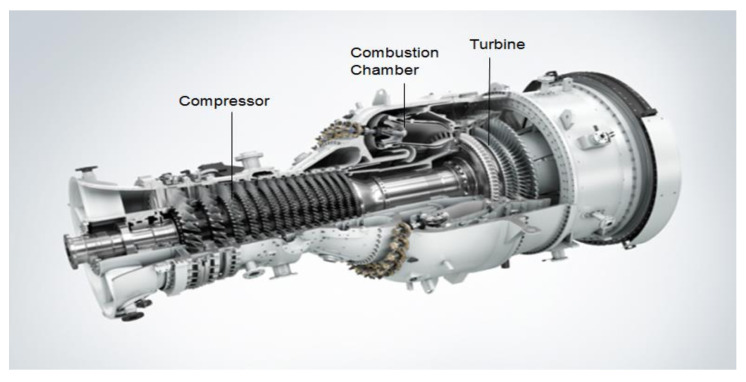
General components in a gas turbine (Siemens Industrial Turbomachinery AB, 2015) [36].

To produce mechanical power, a gas turbine works by compressing the fluid, which in most cases is air, into a compressor and burning the fuel in the combustor, hence increasing the temperature and letting the fluid expand through a turbine, generating energy. These components are depicted in Figure 1. Finally, the temperature and pressure are simultaneously reduced. By the end of the process, the mechanical power is transferred to the application [35].

Gas turbines containing these components are known as simple cycle gas turbines. Additional equipment can be added to increase the efficiency of the unit or increase its output. These are technically termed complex cycle gas turbines, which involve extra components [4]. The industrial gas turbines examined in this research work are single-shaft simple cycle systems. A single-shaft turbine is linked to the compressor via rigid coupling. Indeed, the turbine ensures the rotational drive of the compressor to convey a process gas.

### 1.2. Operational Modes

Machine health and safety are determined by condition monitoring. By avoiding unnecessary maintenance actions, costs can be reduced greatly. Consequently, detecting the operational mode of a gas turbine in an industrial process is important since different characteristics of gas turbine operation can be identified for a given industrial process based on the available data. Depending on the nature of each industrial process, the operational modes detected may differ from the expected ones from an engineering perspective due to a number of factors, such as special design conditions, ambient features, and control features.

In defining and determining operational modes, many variables can be taken into consideration. Industrial gas turbines operational modes are usually defined based on the *active load*, that is, the expected energy production. In this sense, *full load* refers to a mode in which a gas turbine is operating at full capacity, delivering as much power as possible under current working conditions. The operational mode of *partial load* refers to any operational mode of working in an intermediate level of energy production that is lower than its rated capacity. Finally, the mode *idle* refers to an industrial gas turbine under working conditions, but waiting in a standby state.

At this point, these *theoretical operational modes* are purely defined from a model-based engineering point of view, based only in one variable, the active load. However, it can be observed from sensors data that other variables modify the general behavior of the system, eventually leading to the definition of more accurate *operational modes*, such as ambient temperature, pressure, or humidity.

### 1.3. Clustering as a Data-Driven Technique

Operational modes in industrial gas turbines can be detected using *data-driven* techniques. Operational modes are characterized by some variables showing values belonging to determined ranges and those values being correlated among them. These operational modes are described with some usual values in the relevant variables. Therefore, having available sensor data from all variables in a process, an efficient way of identifying those similar operational modes or states with similar values, is based on detecting homogeneous groups of sensor data at different time stamps. This later process is exactly what a *clustering* method does in an unsupervised dataset.

A data clustering technique is able to automatically determine different clusters of working points. Based on the clustering method at hand, we can find different divisions of operational modes in the system after a proper interpretation process is done. Hence, there is not one solution using data-driven techniques, but several. Obviously, some results are more robust than others. As we cannot identify them in advance, we need to try different methods. However, using different clustering techniques, the problem arises of knowing what is the best partition obtained. One way to solve that issue without making a validation of the obtained partitions, i.e., set of clusters, is by using an *ensemble of clustering* methods [37]. Ensemble of clustering techniques improve the accuracy of the clustering process.

As clustering is an unsupervised technique, no prior information for the pattern is available. In this sense, the cluster ensemble design problem is more difficult than designing classifier ensembles [38]. The clustering techniques ensemble, in several areas, such as machine learning, pattern recognition, bio-informatics, and data mining, is known as consensus clustering, clustering aggregation or clustering combination [39,40]. An ensemble of clustering techniques aggregates multiple partitions of a set of objects into a single joint partition, often referred to as the consensus solution. The cluster ensemble can be used to offer more strong and stable clustering solutions compared to a single clustering method. In fact, cluster ensembles address the issue of combining multiple base partitions of the same set of objects into a single amalgamated partition [37]. Considering that there are a set of partition results, the goal is finding a consensus partition that agrees as much as possible with the given partition results.

Some properties of cluster ensemble are robustness, consistency, stability and novelty. Different clustering algorithms present various benefits and drawbacks. Some of them might perform well in specific datasets but not in others, or they might be very sensitive to parameter settings. By aggregating the results of different clustering processes the robustness and the quality of the final partition will be improved [39]. Moreover, the clustering process aggregation integrates information in order to obtain consistent and strengthen results, and the results are pretty similar to the combination of single clustering algorithms. Furthermore, the given consensus solution of ensemble clustering process is unattainable by implying single clustering algorithms. Furthermore, it is more stable. It defines an appropriate number of clusters, and it can also detect outliers. Therefore, the consensus solutions are lower sensitive to noise and outliers [41].

### 1.4. Clustering Methods

Machine learning methods for data analytics are usually grouped into supervised and unsupervised learning. The latter one refers to the process of learning from unlabeled data. Clustering methods are one example of unsupervised methods. They can be classified into four broad categories: *partitioning algorithms*, *distribution-based clustering*, *hierarchical algorithms*, and *density-based algorithms* [42].

Using a *partitioning* method, instances re-position themselves between clusters by moving from one to the other, starting from an initial partition. This type of clustering usually requires that the user presets the number of clusters in advance. The K-means method is an example of the partitioning method [43].

The *distribution-based* clustering method involves grouping the observations according to their distributions: observations within the same cluster most likely come from the same distribution. The Gaussian mixture model is an example of a distribution-based clustering technique [44].

*Hierarchical* clustering methods create clusters through recursive partitioning of instances in a top-down (divisive methods) or bottom-up (agglomerative procedures) process. Agglomerative hierarchical methods produce a dendrogram that represents the nested grouping of objects and the denominator of similarity levels. Agglomerative hierarchy-based clustering methods can be classified into three main groups based on the method used to calculate the similarity measure: single-link, complete-link, and average-link clustering. Using single-link clustering, the distance between a pair of clusters equals the shortest distance between any member of one cluster to any member of the others. For the complete-link approach, the distance between two clusters is equal to the longest distance between members of the two clusters. In average-link clustering, the distance between two clusters is equal to the distance between any member of one cluster and any member of the other cluster.

*Density-based* methods assume that every point that constitutes the cluster is drawn from a specific probability distribution. Several distributions are assumed to conform the overall distribution of the data. DBSCAN and HDBSCAN are both examples of density-based methods. DBSCAN (density-based spatial clustering of applications with noise) is very popular because it successfully locates clusters of arbitrary shapes on large spatial databases. By searching the neighborhood of each object in the database and checking whether it contains more than the minimum number of objects, the algorithm finds clusters.

In order to automatically detect operational modes, we would like to link data-driven clusters with them. We propose to use an ensemble approach that provides robust clusters and can therefore be able to identify accurate, unknown and reliable system operation modes.

The rest of the paper is organized as follows. In Section 2, the materials and the methods for the proposed scheme that identify the operation modes of gas turbine systems for prognostic capabilities are described. Results on real data from Siemens Energy’s medium-sized gas turbines are presented in Section 3. A discussion about the interpretation of the obtained results is presented in Section 4, followed by the conclusions in Section 5.

## 2. Materials and Methods

**Figure 2:**
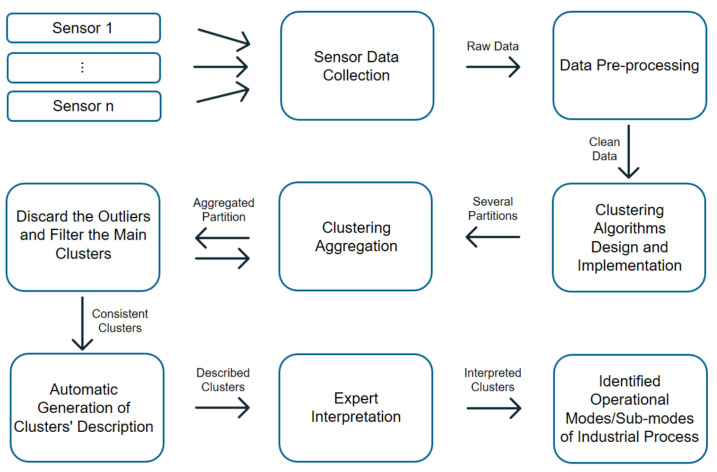
Chart of the proposed methodology pipeline to identify unknown operational modes through an ensemble of clustering methods.

A continuous stream of data from hundreds of variables is continuously collected in industrial systems, such as gas turbines. Changes in the relationships among these variables over time indicate that several operational modes are being observed. Our goal is to develop accurate mechanisms that are simple and efficient, which can be used with rapidly changing industrial data streams. Through the use of clustering techniques, we are able to automatically detect the different operational modes. Each mode is represented as a cluster, and we can identify the operational modes by characterizing these clusters. As shown in Figure 2, the pipeline represents the methodological steps that take place throughout the research process. Firstly, real-world data are collected from the most appropriate sensors, and then these data are preprocessed and fed into different clustering algorithms. At this point, different clustering methods are selected and designed to cover different aspects of clustering. Next, multiple clustering techniques are applied, as well as different types of validation techniques simultaneously, including qualitative validation via expert knowledge and structural validation via cluster validation indices. After the different results from different perspectives are generated, the ensemble clustering technique is applied to aggregate the results. During the next phase, the system is designed to extract different partitions with a control parameter in order to obtain more accurate and robust clusters that correspond to operational modes and sub-modes. This is followed by a stage of filtering to remove unnecessary clusters. At the end of the pipeline, the obtained clusters undergo a conditional distribution analysis by each cluster to generate automatically the description of each cluster. Described clusters are interpreted by domain experts. Finally, the interpreted clusters become the different operational modes discovered from the sensors’ data of the industrial process.

Here, we explore a robust and stable way to achieve consensus partition. Although we use different clustering algorithms here, and describe them in Section 2.1, since clustering is an unsupervised method, it is not obvious how one is superior to another. Therefore, we were not able to determine in advance which algorithm would be optimal to represent various operation modes. This vulnerability of clustering techniques causes uncertainty about which is the good partition. As we are looking for the most consistent clusters to be a good representation of the operational modes, we opted for the use of an ensemble of clustering techniques as the means of having the most consistent result.

### 2.1. Clustering Algorithms

Using a vast amount of historical data from industrial gas turbines, information about the operational modes can be inherited by partitioning the data points into a set of groups which are as similar as possible. We used different methods of clustering for this partitioning.

For clustering data, one of the most frequently used clustering algorithm is *K-means clustering*. As a first step of the K-means algorithm, we select *K* random centers that indicate the number of clusters. Each data point then corresponds to the nearest center based on its distance. In the next step, new centers are computed, and the data points are updated, so the process is repeated until no clusters differ between iterations.

*Gaussian mixture models* (GMMs) are a powerful probability-based tool for clustering, which can be seen as an extension of the ideas behind K-means. In this approach, the centroid (mean), the covariance and the cluster size are taken into account when describing each cluster. GMMs attempt to find a mixture of multi-dimensional Gaussian probability distributions that align with an input dataset [45].

A *hierarchical clustering* technique produces a tree relationship between clusters, i.e., *a dendogram*. Hierarchical clustering is accomplished by two basic algorithms. While one algorithm depicts the relationship from the bottom up (agglomerative or merging), the other algorithm describes the relationship from top to bottom. In the agglomerative method that we used in our study, each observation is assigned to a separate cluster. Next, a distance between each cluster is calculated and so the two clusters with the best match are merged. At the end, the last two steps are repeated until only one cluster remains [46].

*Density-based clustering* methods are widely used for a number of reasons, including robustness to noise or not requiring the number of clusters as an input parameter. As a result of these benefits, it is increasingly popular as a powerful clustering method. In this paper, we applied DBSCAN and HDBSCAN (Hierarchical DBSCAN) algorithms to find clusters in the dataset in order to discover different operational modes in gas turbines. As a density-based clustering algorithm, DBSCAN defines a cluster as a dense region of points that is separated from the other points by low-density regions. A general idea in DBSCAN is to keep growing the cluster as long as the density in the neighborhood exceeds a certain threshold. It is theorized that each object within a given cluster must contain a minimum number of points within the neighborhood of a given radius, the epsilon neighborhood. Because DBSCAN cannot handle hierarchical clusters, hierarchical density-based clustering methods, such as HDBSCAN, were developed. There are several advantages for HDBSCAN, including the fact that it does not require to define a priori the number of clusters, as well as being robust to clusters with different shapes and densities. Furthermore, HDBSCAN is reliant on only one hyperparameter, the minimum number of points per cluster. Additionally, the probability of assigning a cell to a cluster is defined for each cell [47].

### 2.2. Data Processing

The data-gathering process in the pipeline begins with data collection. Data from the real world typically have inconsistencies, errors and are probably noisy because of the typical big size of the database. In that case, it is necessary to do some pre-processing in order to obtain a dataset ready for analysis. Pre-processing involves data cleaning, instance selection, normalization, transformation, feature extraction and selection.

The dataset is originated from a medium-sized power gas turbine collected and owned by Siemens Industrial Turbo-machinery (SIT). Therefore, the dataset itself cannot be disclosed due to confidentiality issues. There are a variety of sensors surrounding a gas turbine, which are used to detect its behavior. Data feeding clustering algorithms come from the most interesting sensors, which are selected by experts to represent the gas turbine behavior. In the case of an industrial gas turbine, there would be many valuable signals, such as inlet air flow, inlet and outlet temperature, pressure data from both the compressor and the turbine, and guide vane position. There is also a sensor that detects full load operation as a means to detect the operational mode. Those sensors were tested for reliability and quality, and all of them obtained a good evaluation.

In this situation, *eleven* different characteristics are considered for the clustering approaches. Table 2 provides a description of the feature set taken into account by various clustering methods. The features starting with *C* and *T* are related to the compressor and turbine, respectively. Differential ambient temperature (DAT) describes the difference between the ambient temperature and the compressor’s inlet temperature. CDP shows the difference between the pressure after the filters and the pressure just before letting in the compressor. As a result, a proportional value is obtained of the mass flow of air entering the compressor. The compressor output and compressor inlet temperatures are indicated through COT and CIT, respectively. CIP represents the inlet pressure of the compressor, and CPR is the ratio between the compressor’s outlet pressure and its inlet pressure. One of the most important parameters for estimating the performance of the turbine is the turbine inlet temperature, or TIT. The important aim of turbine research is to increase the TIT without reducing the turbine’s lifetime. The turbine outlet energy or TOE refers to the energy coming out from the turbine. The exhaust temperature of a turbine is indicated by TET. TEDP is the difference in pressure between the exhaust gas from the turbine and that of the ambient air. In GVP, the piston distance is measured as a percentage.

For clustering data, the values of the features are scaled after feature selection. Since value ranges for each feature are so wide and dissimilar, we chose to apply feature scaling such that the data can be compared within a small range on a common scene. Working with unscaled data presents a challenge to visualization and can decrease the accuracy of machine learning algorithms. Thus, feature scaling is an important step in data pre-processing. Among several possibilities, in this paper, we employed standardization and normalization techniques for clustering methods and visualizing partitions, respectively.

Standardization or Z-score normalization transforms data such that the distribution of the features has zero mean and unitary standard deviation. Hence, for univariate data *X* with *N* samples $x_{i}$, standardized *X* is defined as follows [48],

(1) $$X_{standardized}=\frac{X-\bar{X}}{\sigma (X)}$$

with mean value

(2) $$\bar{X}=\frac{1}{N}\sum_{i=1}^{N}x_{i}$$

and standard deviation

(3) $$\sigma \left(X\right)=\sqrt{\frac{1}{N}\sum_{i=1}^{N}{\left(x_{i}-\bar{X}\right)}^{2}}$$

In min-max normalization, the feature is linearly rescaled in the range 0–1 [48]:

(4) $$X_{normalized}=\frac{X-\min(X)}{\max(X)-\min(X)}$$

Next, a data-cleaning process was performed. This dataset does not include missing values, and hence, no missing value treatment is needed. Regarding the abnormal values, either error values or possible outlier values are managed accordingly. The error values are checked, but no error values are found. Next, an outlier detection method is implemented to detect, and occasionally, to eliminate, outlier elements. A winsorized process is employed to limit the effects of outliers by limiting extreme values in the data since classical statistics, such as the mean and standard deviation, are sensitive to extreme values. Through winsorization, the impact of extreme observations is reduced in order to remove this sensitivity. This is an averaging method in which the smallest and largest values are replaced with those closest to them. GVP and TOE are changed under this regime.

Finally, we made use of some dimensionality reduction methods to visualize the obtained clusters. As the dataset contains too many features, it becomes difficult to visualize them, so reducing the dimension of the dataset to 2D allows us to visualize patterns more clearly. Dimensionality reduction can be accomplished via several techniques. The main techniques include random projection techniques, linear dimensionality reduction methods, and nonlinear dimensionality reduction or manifold learning methods. Taking a random projection of the data is the simplest way to reduce dimensionality. A random projection, however, is likely to obscure the more interesting information in the data. Hence, during this study, we used t-distributed stochastic neighbor embedding (t-SNE) and uniform manifold approximation and projection (UMAP) as nonlinear methods, along with principal component analysis (PCA) as a linear method.

**Figure 3:**
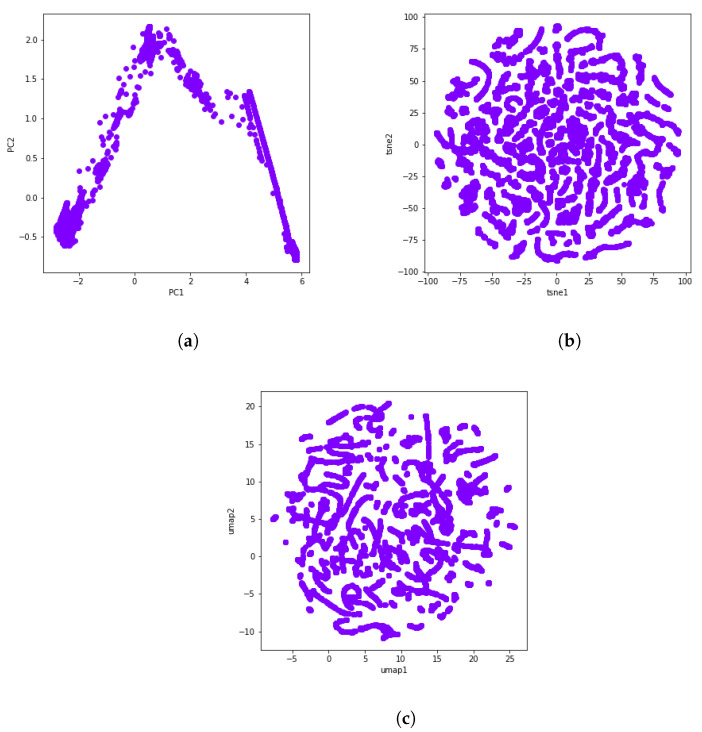
A visual representation of PCA, t-SNE, and UMAP through the use of data from the gas turbine GT281 during 16 days. (**a**) PCA. (**b**) t-SNE. (**c**) UMAP.

During PCA, data are rotated and mapped along the direction of increasing variance in order to find the subset of variables with the maximum variation that can be measured. These are the principal components [49]. Figure 3a shows the dimensionality reduction result of the selected dataset using two principal components.

The new cutting edge t-SNE technique is modeled as a stochastic neighbor embedding (SNE). It keeps both the local and global structures of the original data, and preserves well-separated clusters [50]. UMAP is one of today’s best dimensionality reduction methods and is very similar to t-SNE. Like t-SNE, UMAP also uses a graph layout algorithm. In this sense, the UMAP algorithm relies on algebraic topology and Riemannian geometry. Though both techniques provide a similar output, UMAP often arguably preserves the global structure better in the final projection, i.e., the inter-cluster distance is more meaningful with UMAP than with t-SNE [51]. The t-SNE and UMAP visualizations can be seen in Figure 3b and Figure 3c, respectively.

### 2.3. Software and Hardware

In this study, the proposed method was developed in Python 3.6.0 programming language [52], and for implementation and visualization, we used a variety of libraries, mainly including Scipy 1.1.0 [53], Numpy 1.18.0 [54], Scikit-Learn 0.20.0 [55], OpenEnsemble 1.1.1 [56], hdbscan 0.8 [57] and UMAP 0.4.6 [58].

The hardware used to run the software was the Amazon SageMaker service with 2 CPUs and 8 MGB of memory. Notwithstanding, a powerful laptop can be also used, but with higher computational cost.

## 3. Results

The key result searched out in this study is the identification of industrial gas turbine operational modes by means of a clustering ensemble approach that aggregates the information obtained from several clustering techniques.

### 3.1. Dataset

In this work, analyses refer to data collected by Siemens Energy and concern one of the medium-sized types of gas turbines. Sensors are placed at different locations inside the gas turbine to collect data. In this study, a 1-min sample time dataset $X\in R^{n\times d}$ was analyzed, with n= 23,000 data points (almost 16 days) and d=11 eleven sensors or features taken into consideration.

**Figure 4:**
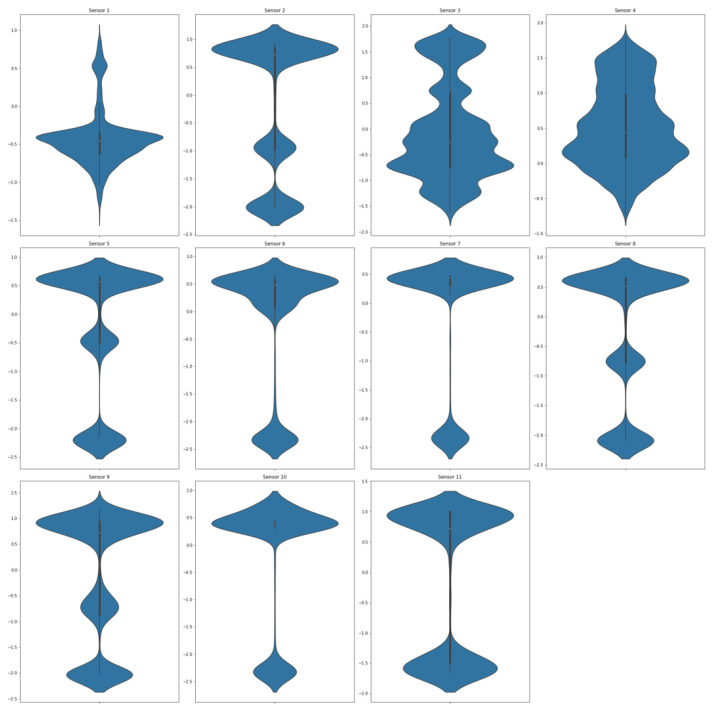
Violin plot of the normalized selected dataset for each of the features. The white dot in the middle represents the median value, and the thick black bar in the middle represents the interquartile range. The thin black line extending from it represents the upper (max) and lower (min) adjacent values.

Due to the importance of detecting the transitory effects of operational modes in a gas turbine, the absolute minimum requirement is that datasets are of 1-min length. We selected this dataset so that we could capture a period of time with the maximum amount of output power fluctuation, which is the most important indicator for a gas turbine’s operation states. In Figure 4, the violin plot displays a summary of the statistical description of the data.

**Figure 5:**
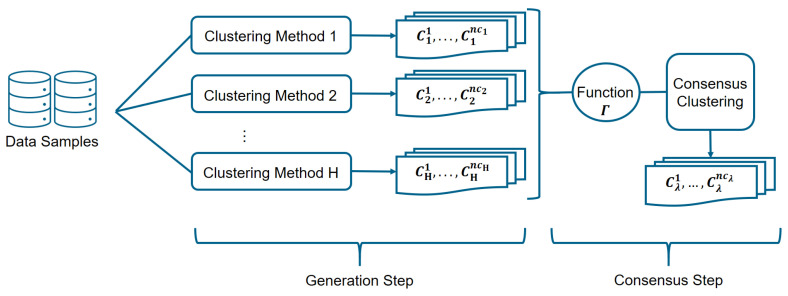
The clustering ensemble algorithm diagram shows the clustering aggregation process.

Two steps are required to detect the operational modes of the gas turbine by means of the proposed ensemble of clusters method, as it is shown in Figure 5. Results from some independent and diverse runs of clustering algorithms are stored in the first step, which is called the *generation step*. Next, in the *consensus step*, the final partition (consensus clustering) is determined by combining those schemes using a specific consensus function Γ [37].

### 3.2. Cluster Generation Step

As it is impossible to know in advance which clustering algorithm will be appropriate for the problem at hand, in the *generation step* of the cluster ensemble framework, several data partitions are produced for aggregation in order to optimally provide a consensus partition in a robust and stable manner [59]. As shown in Figure 2, we start the ensemble clustering pipeline by designing and selecting some clustering algorithms. Next, clustering ensembles are produced. They can be generated by using several techniques: (i) employing different clustering algorithms, (ii) implementing the same clustering algorithm with several hyperparameters or initialization parameters, (iii) exploring different dissimilarity measures within a given clustering algorithm, (iv) using different sets of data objects, (v) selecting distinct subsets of data features, and (vi) projecting the original data space into a lower dimensional data space [60,61,62,63].

In this research, the diversity of clustering is provided as follows. Clustering techniques of four kinds are implemented: K-means is applied as a partitioning clustering method, Gaussian mixture models (GMMs) as a distribution-based clustering method, agglomerative algorithm for hierarchical techniques, and DBSCAN and HDBSCAN for density-based clustering methods. To generate different partitions after considering different methods of clustering, a variety of initialization parameters and hyperparameters selection are examined. For instance, the K-means algorithm is applied for each data partition using the different number of clusters within the [3,10] interval. For the DBSCAN and HDBSCAN approach, the epsilon neighborhood uses epsilon={0.75,0.65,0.6,0.55,0.50}, the minimum number of points within the radius min_point=22 (2 × number of features), and min_samples={100,120,150} are considered. Moreover, for the agglomerative technique, the average, complete, and ward linkages are applied. Some of the dissimilarity measures used in clustering methods are Euclidean and $l_{1}$.

**Figure 6:**
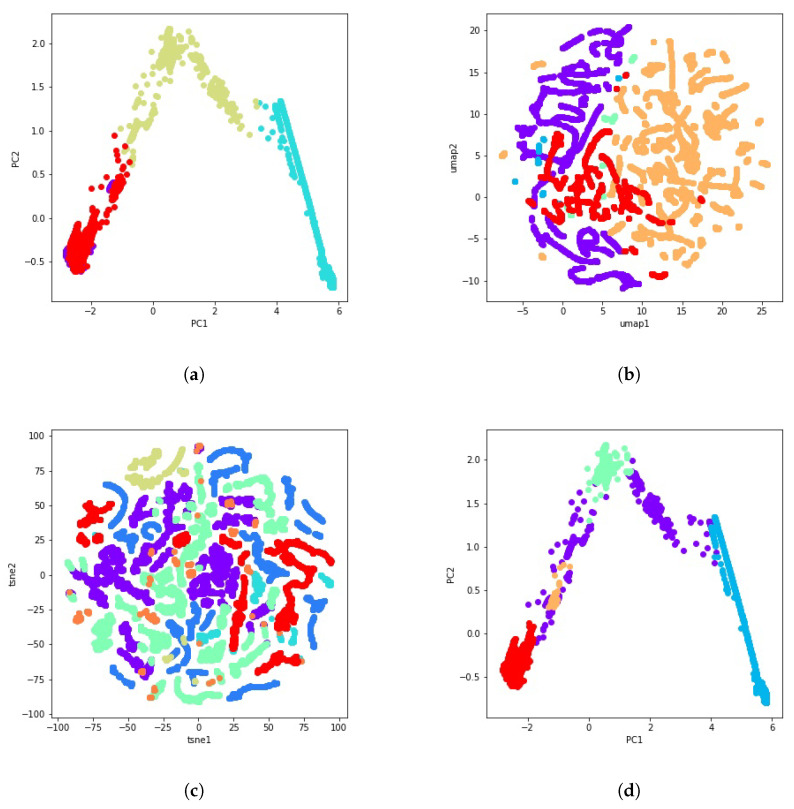
Some examples of the 51 calculated clustering solutions using PCA, t-SNE and UMAP. (**a**) PCA visualization of the K-means clustering result with 4 clusters. (**b**) UMAP visualization of the agglomerative clustering result with average linkage and Euclidean distance. (**c**) t-SNE visualization of the GMM clustering result with 3 components. (**d**) PCA visualization of the HDBSCAN clustering result with min_samples=150.

In our work, a set of H=51 partitions of the dataset *X* is generated. Hence, each point $x_{i}$ is assigned to a set of labels:

(5) $$x_{i}\to \left\{L_{1}^{i}=\pi_{1}\left(x_{i}\right),\ldots ,L_{H}^{i}=\pi_{H}\left(x_{i}\right)\right\}$$

where $L_{j}^{i}=\pi_{j}\left(x_{i}\right)$ denotes the label assigned to $x_{i}$ by the *j*-th clustering algorithm $\pi_{j}$ [64]. Consequently, with the set of *H* partitions, it can be defined:

(6) $$\begin{array}{ccc} \Pi & = & \left\{\pi_{1},\pi_{2},\ldots ,\pi_{H}\right\}\\ \pi_{j} & = & \left\{C_{j}^{1},\ldots ,C_{j}^{{nc}_{j}}\right\}\\ X & = & C_{j}^{1}\cup \ldots \cup C_{j}^{{nc}_{j}}\;,\;\forall \pi_{j} \end{array}$$

with ${nc}_{j}$ being the number of clusters in the *j*-th partition and $C_{j}^{k}$ the set of points in the *k*-th cluster of partition *j* [65]. Figure 6 illustrates some examples of these 51 clustering solutions using PCA, t-SNE, and UMAP for visualization.

### 3.3. Establishing Consensus

The *consensus step* defines how the multiple data partitions are combined into one consensus partition. There exist two main consensus function approaches: *objects co-occurrence* and *median partition*. In the first approach, it is analyzed how many times an object belongs to one cluster or how many times two objects belong together to the same cluster. In the second approach, the consensus solution is theoretically gained by solving an optimization problem about finding the median partition by maximizing the similarity measure between all the partitions in the cluster ensemble [66].

**Figure 7:**
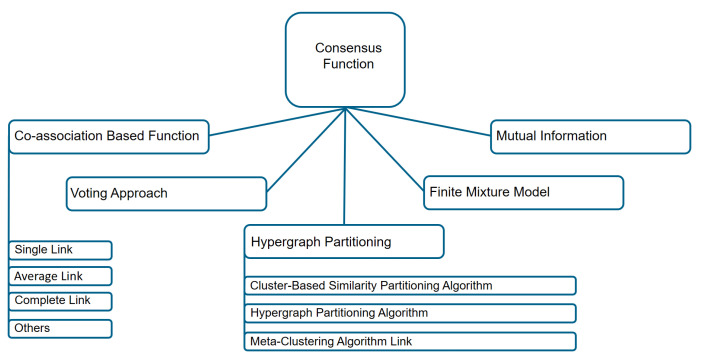
Diagram of the consensus functions techniques based on object co-occurrence approach.

Among the methods for the consensus step, co-association based methods, hypergraph partitioning based methods, methods based on mutual information, methods using finite mixture models and voting based methods are the most common ones (see Figure 7). Using a co-association function, one tries to map partitions, which are observed in the cluster ensemble, into a matrix, whose values represent how many times these objects are found in the same cluster [61]. With graph-based methods, ensemble partitions are converted into a graph or hypergraph representation problem [38]. It consists of vertices and hyperedges, where vertices represent data points and hyperedges describe a set of objects belonging to the same clusters. Mutual information is carried out as a similarity measure between partitions, using mutual information maximization between labels of the partitions in the cluster ensemble, and labels of the consensus partition [67]. Using the expectation maximization (EM) algorithms, a finite mixture model approach maximizes a likelihood estimation problem. As a result, a solution to the maximum likelihood problem will give us a consensus partition [64]. Relabeling and voting methods are based on how many times each object belongs to a cluster. In this case, the consensus partition is reached through the voting process after labeling the clusters through the voting process [37].

In general, the objective function for a cluster ensemble can be described by a consensus function [38], $\Gamma :\Pi \subset N^{n\times H}\to \Lambda \subset N^{n}$ to represent an integration of clusters,

(7) $$\Gamma :\{L_{\left\{1,2,\ldots ,H\right\}}^{i}=\Pi \left(x_{i}\right)|i\in \left\{1,2,\ldots ,n\right\}\}\to \Lambda$$

The above formula defines the consensus function Γ for a set of *H* given clustering solutions (a set of labels), with the *j*-th grouping $\pi_{j}$ inducing clusters $C_{j}^{k}$. Here, the objective is to find the best partition $\Lambda =\{C_{\lambda }^{1},\ldots ,C_{\lambda }^{{nc}_{\lambda }}\}$, inducing new clusters for data in *X* out of a set of *H* partitions, Π. Our paper focuses on finding consistent clusters within a set of partitioned data by using *majority voting* methods. Combining the results of the different clustering algorithms, due to their different strengths and weaknesses, if they work together, their contributions are expected to compensate each other.

In a clustering algorithm, neighbor data objects within a cluster are more likely to be co-located in the same group. According to the partitions of the data derived from various clustering methods, pairs of data points receive a vote for association in each separate run. Accordingly, an obvious idea to identify the data points which are repeatedly assigned to the same cluster is the construction of a pairwise co-association matrix, that is, a matrix S in which each (i,j) cell represents the percentage of times a given data object pair has co-occurred in a cluster:

(8) $$\begin{array}{c} S_{ij}=S\left(x_{i},x_{j}\right)=\frac{1}{H}\sum_{k=1}^{H}\delta (\pi_{k}\left(x_{i}\right),\pi_{k}\left(x_{j}\right)),\\ \delta \left(a,b\right)=\left\{\begin{array}{cc} 1, & \begin{array}{cc} if & a=b \end{array}\\ 0, & \begin{array}{cc} if & a\neq b \end{array} \end{array}\right. \end{array}$$

As a result, the co-occurrence of each event is equivalent to a vote in favor of their coming together as a cluster. By dividing this matrix by the number of clustering solutions *H*, a normalized voting result is obtained, given that each co-occurrence is a vote toward their gathering in a cluster. A majority vote is used to generate the underlying data partition in which normalized votes are compared with a specific threshold, and all data linked this way are grouped together in the same cluster. It can be said that this threshold represents a *consistency threshold*, a number in the range [0,1]. By thresholding the co-association matrix, as previously described, it should be possible to recover a robust cluster structure and their consistent clusters. In this case, when the threshold is set, the *cluster consistency*, which is a percentage, is measured as 100 times the consistency threshold.

**Figure 8:**
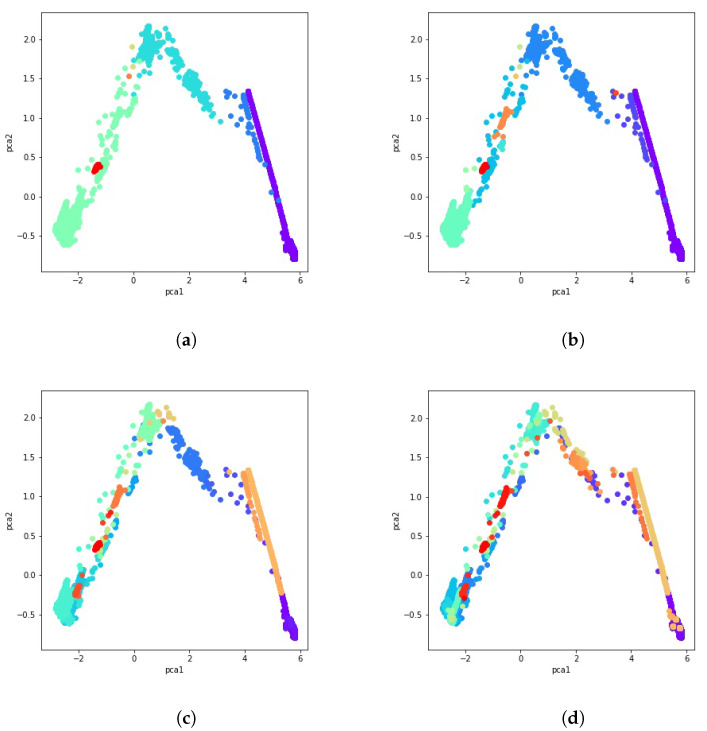
PCA visualization of the ensemble of clusters with several percentages of consistency. (**a**) 80% consistency. (**b**) 85% consistency. (**c**) 94% consistency. (**d**) 98% consistency.

After calculating the co-occurrence matrix for these 51 clustering solutions, a data cluster is created by aggregating data with a majority vote and comparing normalized votes with the fixed threshold. Results obtained using the majority vote algorithm as the ensemble of clusters method are presented in Figure 8a through Figure 8d. Different consistency thresholds are taken into account, and the results are illustrated using a 2D PCA visualization. For instance, the consistency threshold 0.8 means at least 80% of the clustering techniques vote for that data point to be part of that cluster, that is, an overall cluster consistency of 80%.

### 3.4. Structural Clustering Validation

**Figure 9:**
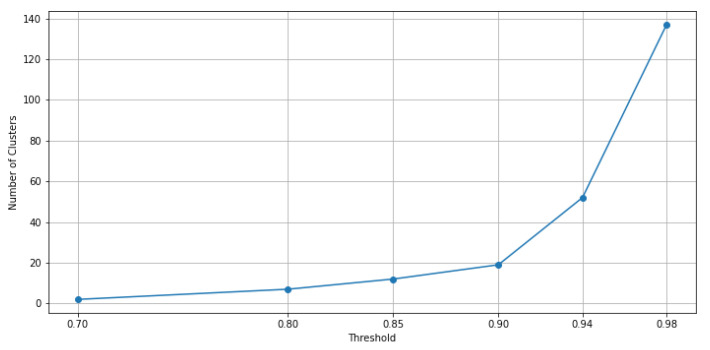
Graph of the number of clusters derived using the ensemble of clustering methods when the consistency threshold is increasing.

It is worth noting that by increasing the consistency threshold for the majority votes method, more clusters are obtained. Based on different values of this so-defined consistency threshold, it is represented in Figure 9 how the number of clusters increases according to this threshold for the considered data.

**Figure 10:**
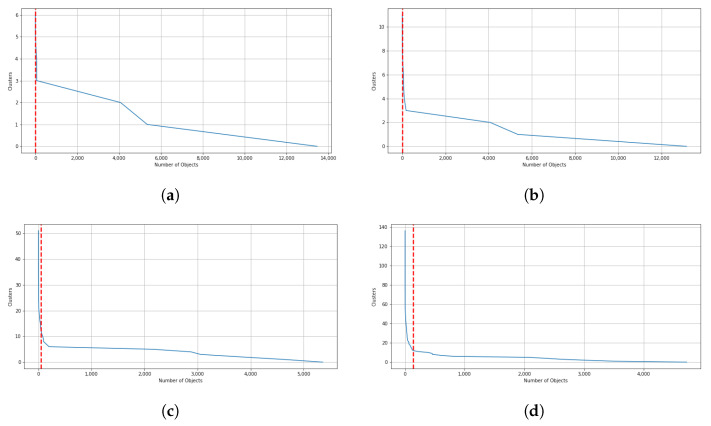
In this illustration, filtering values based on different consistency of cluster in ensemble clustering are demonstrated by red lines. (**a**) 80% consistency of clusters. (**b**) 85% consistency of clusters. (**c**) 94% consistency of clusters. (**d**) 98% consistency of clusters.

**Figure 11:**
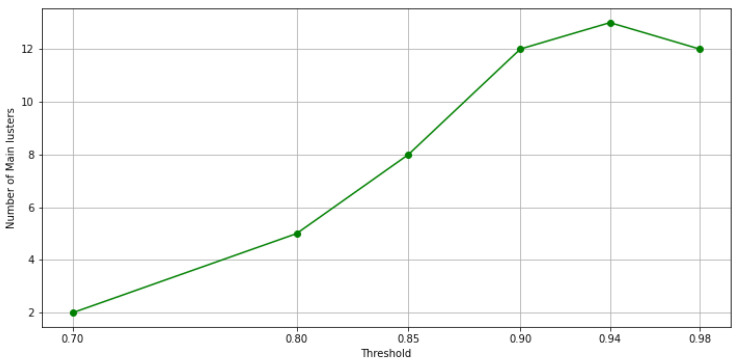
The number of main clusters in each ensemble solution is shown in this figure, based on the filtering method.

**Figure 12:**
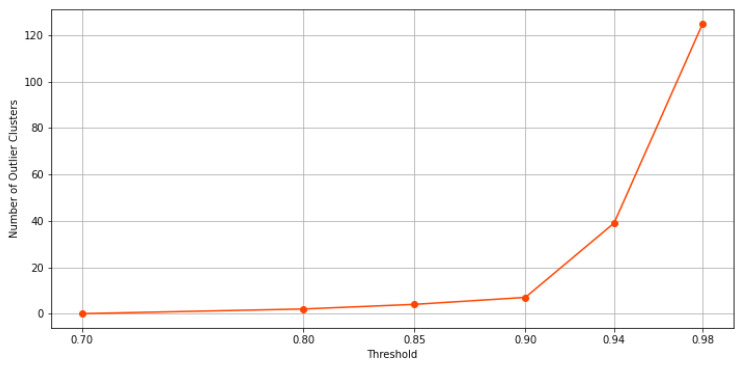
This figure shows, according to the filtering method, how many clusters in each ensemble solution are potentially outliers.

When the threshold value increases, and thus, the exigence to belong to the same cluster for the data point, among these clusters, some possible outlier clusters appear. For example, if a cluster only contains 1 data point, that would definitely be an outlier cluster. To address this issue, we propose a filtering method designed to extract main clusters and discard outlier clusters, which is similar to the elbow method. In this method, the elbow on the curve represents the minimum amount of objects or data points that must be within each cluster of data. This is known as a *filtering value*. The clusters whose data points fall below this value are considered outlier clusters. As a result, clusters with a number of data points below this value are automatically assigned to the main cluster. In Figure 10a through Figure 10d, the results of the filtering criteria on ensemble clustering with different consistency thresholds are depicted. Based on this method, the filtering values of the ensembles’ results with 98%, 94%, 90%, 85%, 80%, and 70% consistency of the clusters are 136, 51, 18, 11, 6, and 0, respectively. As an example, if the ensemble results are consistent with 98%, the main clusters are constructed by over 136 data points, and if the ensemble results are consistent with 70%, the clusters are all main ones. Figure 11 and Figure 12 show the number of main and outlier clusters inside each ensemble solution as determined by the filtering method, respectively. Hence, the number of main clusters in ensemble solutions with 98%, 94%, 90%, 85%, 80%, and 70% cluster consistency are 12, 13, 12, 8, 5, and 2, respectively. Considering that the cluster consistency factor contributes to cluster formation, Table 3 gives a summary of the number of clusters formed. Following this, we analyze the statistical data of the main clusters and interpret the results, and then determine which clusters represent which modes of operation in the gas turbine.

## 4. Discussion

**Figure 13:**
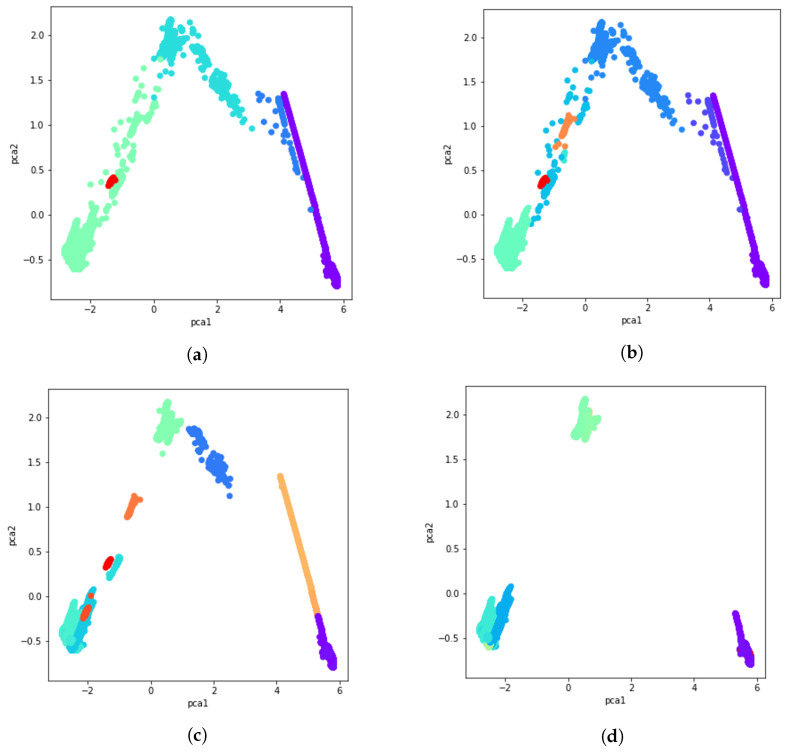
Following the filtering process, the PCA visualization of cluster ensembles with different cluster consistency is shown. (**a**) With 80% consistency of clusters. (**b**) With 85% consistency of clusters. (**c**) With 94% consistency of clusters. (**d**) With 98% consistency of clusters.

As a result of incorporating different clustering methods and aggregating the extracted information and validating it with the proposed filtering method, we obtain a final result which should match some operational modes. Following the filtering methods applied for different consistency rates, the final results of the ensemble of clustering techniques are shown in Figure 13. However, how can we interpret these operational modes, and how can we determine in what operational mode the gas turbine is operating? Our explanation can be made first with the help of statistical techniques based on the observation of the conditional distribution of variables for each cluster. This analysis leads automatically to a characterization of the clusters based on the range of values of the relevant variables. These variables, DAT, CPR, TIT, TOE, and GVP, are the features selected for the characterization and visualization of the clusters. From these described and characterized clusters, the experts make an interpretation of these operational modes.

**Figure 14:**
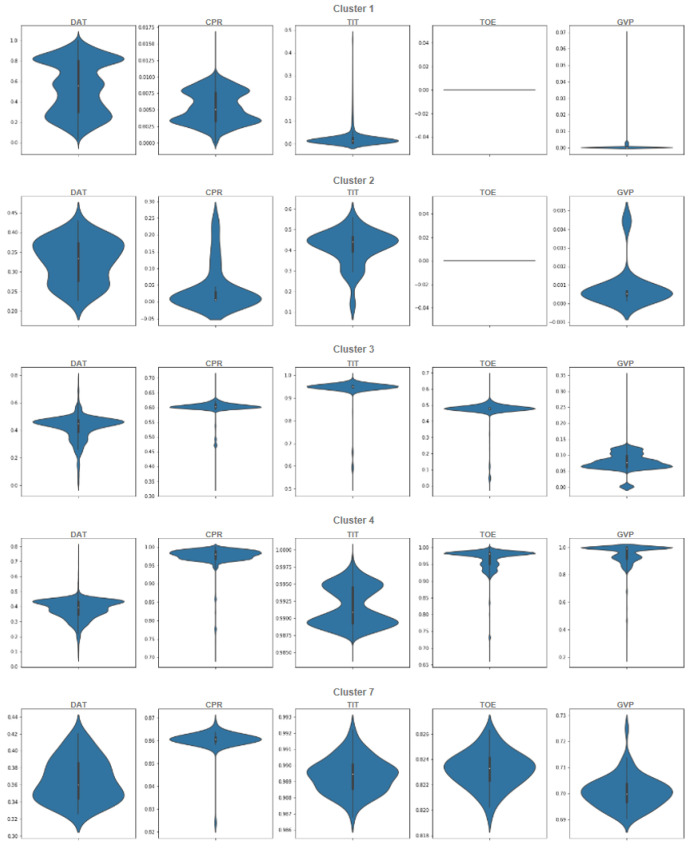
Violin plot of each individual cluster in the ensemble of clusters, with a cluster consistency of 80% for the normalized selected features.

We start analyzing the ensemble of clusters with the 0.80 threshold, which generates five ensemble clusters (see Figure 13a). Even though the ensemble of clusters obtained with the 0.94 threshold seems to be the best one according to the *filtering value*, extracting 13 main clusters, it generates many outlier clusters (39), which impedes the interpretation process; thus, we focus on the 0.80 threshold, which generates 5 main clusters and only 2 outlier clusters. However, the ensemble of clusters obtained with the 0.94 threshold will be analyzed for ensuring consistency among the obtained main clusters. The following violin plots of normalized dataset are provided for better analysis and understanding of the data. In light of Figure 14, the interpretation and determination of the partitions can be performed to determine what kind of operational mode they belong to. Based on the statistical description, *Cluster 1* has 5342 data points, and it is obvious in Figure 14 that all the features are zero most of the time and that GVP takes a value almost close to zero. According to the statistical description, it can be categorized as the operational mode *Idle*. Next, 59 data points make up *Cluster 2*, which is similar to *Cluster 1*, except that values in DAT and TIT are less than a half. It can serve as a *transition* operational mode for the gas turbine. Moreover, there are 4070 data points in *Cluster 3*, which is categorized as a *partial load* because the GVP is almost completely open, and other features are either near the maximum value or are in a middle value. *Cluster 4*, grouping 13,641 data points, is clearly the *full load* operational mode since all of its features are in the highest value, including TOE, GVP, CPR, and TIT. Finally *Cluster 7*, with 65 data points, is again a *transition* operational mode almost like *Cluster 4*, but with a minor difference, which here is marginally less than the previous cluster. *Cluster 5* and *Cluster 6* are outlier clusters with less than six points in each one. These interpretations of the clusters as operational modes are summarized in Table 4.

As stated in Table 3, we started with a consistency rate of 70%, which just shows that the data are separated into two clusters, according to whether or not the gas turbine has output energy. By increasing the consistency rate to 80%, five data clusters appear, as shown in Figure 13a, where two of them show the condition where the gas turbine does not have output energy, or in engineering terms, it is idle, which can be shutdown or started, and another one shows the transition between the not operating and operating conditions. The remainder represents the case when the gas turbine has an active output power, where two clusters show a partial load and full load, and one is on the transition mode.

A higher consistency rate of 85% is achieved by splitting the full load operational mode into multiple partitions or clusters. In Figure 13b, same as Figure 13a, we see two clusters in idle and transition modes, respectively, and two in partial load along with its transition. Four clusters remain, of which three of them pass three levels to reach full load operational mode. With the consistency rate increased to 94%, not only is this splitting accomplished in full load operation modes, but it is performed in idle, partial load, and transition operation modes as well. In this sense, as it can be seen from Figure 13c, only representative data points are left to form the main clusters, with the others discarded as outliers. Upgrading the consistency threshold to 98% (see Figure 13d) causes overfitting and we lose data points that are informative. Actually, by checking both, Figure 11 and Figure 12, we can identify the optimal consistency rate in points of inflection on these curves, which is 94%.

Our main aim in this work was to provide a reliable solution for the detection of *unknown operational modes* in an industrial process from its sensors’ data. This goal was the outcome of the analysis conducted in the Introduction section and summarized in the Table 1, where main similar works were analyzed. Those analyses showed that the existing approaches are biased a priori to discover a given number of operational states, or just to determine how many of them exist or are only focusing in one operational mode. Our proposed methodology can discover the existing operational modes, in general, without using any a priori information, as illustrated by the case study on the gas turbine process. Therefore, it can be stated that the issue we were aiming to address is satisfactorily solved.

Our proposed methodology has several advantages regarding the other similar studies analyzed from the literature. Our approach can discover unknown operational states, and the other approaches cannot. It can be also used to determine the number of operational states, and after having identified the operational modes, a predictive model can be generated according to the clusters’ description generated. In addition, the methodology is able to discard outlier clusters and discover consistent clusters through the consistency threshold value and the use of an ensemble of clustering techniques. Furthermore, our approach can discover the operational modes in a semi-automatic way from the sensors’ data. The characterization of aggregated clusters is done automatically using a conditional distribution analysis of relevant variables in each cluster, and human intervention is only needed in the final step for interpreting the characterization of clusters. Finally, the general nature of our proposed methodology enables it to be used in whatever industrial process. Regarding the limitations of our approach, it can be mentioned that the use of an ensemble of clustering techniques increases the computational cost of the approach against other studies that use only one clustering technique. Furthermore, the intervention of human experts is needed in at the last step of the methodology to provide the final interpretation of the clusters, which will become the identified operational modes of the process. Naturally, as the approach is data driven, a representative dataset from the sensors’ data is needed to feed the whole process.

From the point of view of *cost–benefit*, the proposed methodology is feasible to be implemented and used in any industrial process. The clear benefit is the better ontological understanding of the given process, Furthermore, with some further research based on the operational modes found, some predictive maintenance or early anomaly detection techniques can be implemented, which can save a lot of money for the owners of the industrial process. Regarding the costs, there are not much: in addition to the ones related to the development stage, both the software engineer salary and computation cost are associated. In our approach, no new sensor data are needed. Just the available data coming from sensors’ process are used, and the methodology can identify and detect, with the participation of process experts, the operational modes of the process. There will not be extra maintenance costs because the system can be installed in the usual existing powerful computer system devoted to the management of the industrial process. Therefore, clearly, the benefit obtained, both economic and ontological, are higher than the limited costs of development.

A *practical guideline* for interested readers to apply the proposed approach can be elaborated following all the steps of our proposed methodology:

- Gather sensors’ data available.
- Pre-process the dataset.
- Select and implement clustering algorithms to obtain several partitions.
- Aggregate the partitions through an ensemble of clustering techniques.
- Using a consistency threshold, discard the outlier clusters and obtain the main consistent clusters.
- Generate the automatic description of the clusters through the analysis of the conditional distribution by each cluster.
- Provide the description of the clusters to the experts for its interpretation.
- Take the experts’ cluster interpretation as the identified operational modes of the process.

## 5. Conclusions

The purpose of this study was to develop a general data-driven machine learning approach to discover the possible operational modes of industrial processes. The proposed methodology is illustrated in a case study of a gas turbine process. We seek to develop simple, efficient mechanisms that are accurate and applicable to the rapidly evolving industrial data streams. Through the combination of clustering techniques, it is studied how to detect these operational modes semi-automatically.

This methodology solves the main limitations of similar studies in the literature, which were biased with some previous information regarding the number of operational modes to be discovered, or the type of operational modes to be obtained. The proposed approach can detect and identify unknown operational modes without using any a priori knowledge. *Our approach is completely general for any industrial process* and, furthermore, for any other process where the available data can reveal some operational states. Our research is based on data-driven models because we want a general solution that can be applied to any type of process, whether it be a gas turbine, steam turbine, or whatever process in general. As it is shown in the methodology chart depicted in Figure 2, all the involved steps are completely independent, both from the dataset used in the approach and the industrial process targeted. Thus, the generalization capability of the proposed approach is ensured.

The proposed general methodology is a semi-automatic data-driven approach to detect and identify unknown operational modes from sensor data of an industrial process. The approach only requires human intervention for the final interpretation of the aggregated clusters. These clusters are obtained as the result of a consistency filtering process of the aggregated clusters obtained from an ensemble of clustering techniques.

The discovered operation modes are significant to characterize how the process is being operated and give a high amount of information to the managers of the industrial process on how to control and manage it.

Another important outcome of our work is that in addition to the general methodology proposed, a practical guideline for interested readers is elaborated, as described in the previous section.

### Future Work

After the proposal of our methodology for discovering and defining these operational modes, in the future, we will develop a transition network model based upon these operational modes. The sequence of operational modes clusters will allow us to make predictions about the performance of different parts of the gas turbine, using patterns extracted from the data. Following this, we can investigate the impact of operational state clusters and their transition on the performance of the component in order to develop a predictive maintenance model. Among the potential future directions of research, another one is to use recent findings on the application of deep learning techniques, such as [68,69,70]. In the same form that they are usually applied to computer vision recognition, it is also possible for them to be applied to clustering. Most of the works proposing the use of deep learning in clustering tasks are within the same domain of image analysis (image segmentation), such as that described in this review work [71]. Notwithstanding, the idea of using convolutional neural networks (CNN) for dimensionality reduction and to transform the original sensor dataset space into a latent space can be interesting. In the latent space, the dimensions could be reduced and/or different, making the clustering process easier than in the original data space, as described in the survey of [72].

## Figures and Tables

**Table 1:** Survey of similar studies on operational states/modes detection in the literature.

Article	Overview	Method	Limitation
Liu et al. [26]	Diagnoses the 8 most common faults seen in photovoltaic arrays.	Gaussian kernel fuzzy C-means clustering algorithm	The number of different states is a priori known.
Tejedor et al. [27]	Detects and classifies threats and identifies activities.	Several techniques such as Gaussian mixture models	Classify new data into well-defined operational states.
Pan et al. [28]	Based on electrocardiogram signals, identifies a pilot’s fatigue status.	Learning vector quantization (LVQ) and support vector machines (SVM)	
Wu et al. [29]	Identifies the operating state of converter transformer.	Deep belief network optimization algorithm	
Simon and Litt [30]	Extracts steady-state engine operating points.	Mean and standard deviation calculations are combined with domain-specific logic constraints.	Only focusing on one mode of operation.
Davison and Craig [31]	Measures how close an engine is to steady state during operation.	Estimates the rate of change of the assessed parameter and variation about that change	
Celis et al. [32]	Identifies the operating regimes of industrial gas turbines, and monitors and diagnoses steady state conditions during operation.	Moving window approach	
Mikielewicz et al. [33]	Analyzes the partial load of gas turbines.	Based on theoretical analysis on micro turbines	
Zhang et al. [34]	Determines the number of operational modes represented by clusters.	Multivariate statistical process control data (MSPC)	Determine only how many modes of operation there are.

**Table 2:** Feature set used for clustering.

Name	Description
DAT	Differential Ambient Temperature
CDP	Compressor Differential Pressure
CIP	Compressor Inlet Pressure
CIT	Compressor Inlet Temperature
CPR	Compressor Pressure Ratio
COT	Compressor Outlet Temperature
TIT	Turbine Inlet Temperature
TOE	Turbine Outlet Energy (Active Load)
TEDP	Turbine Exhaust Differential Pressure
TET	Turbine Exhaust Temperature
GVP	Guide Vane Position

**Table 3:** A summary of the number of clusters formed by considering different cluster consistency factors.

Cluster Consistency	# Clusters	Filtering Value	# Outlier Clusters	# Main Clusters
70%	2	0	0	2
80%	7	6	2	5
85%	12	11	4	8
90%	19	18	7	12
94%	52	51	39	13
98%	137	136	125	12

**Table 4:** A summary of the clusters’ interpretation obtained with the 0.80 threshold.

Cluster Identifiers	# Points	Cluster Type	Cluster Interpretation	Operational Mode
Cluster 1	5342	Main	Most values are 0	Idle
Cluster 2	59	Main	Most values are 0, DAT, TIT < 0.5	Transition to/from PL
Cluster 3	4070	Main	Most vars middle values	Partial-Load (PL)
Cluster 4	13,641	Main	Highest values in most vars	Full-Load (FL)
Cluster 5	2	Outlier		
Cluster 6	1	Outlier		
Cluster 7	65	Main	High values in most vars	Transition to/from FL

## Data Availability

Restrictions apply to the availability of these data. Data were obtained from Siemens Energy and are available from the authors with the permission of Siemens Energy.
